# Prognostic factors of meningeal carcinomatosis in breast cancer patients

**DOI:** 10.1590/1806-9282.20240177

**Published:** 2025-08-08

**Authors:** Flávia de Oliveira Lima, Renata Tortato Meneguetti, Auro del Giglio, Felipe José Silva Melo Cruz

**Affiliations:** 1 Brazilian Institute for Cancer Control, Department of Oncology – São Paulo (SP), Brazil.; 2 Faculdade de Medicina do ABC, Discipline of Hematology and Oncology – Santo André (SP), Brazil.

**Keywords:** Breast cancer, Meningeal carcinomatosis, Leptomeningeal neoplasms, Cerebrospinal fluid, Prognostic factors

## Abstract

**Objective:**

This study aims to evaluate the primary signs and symptoms in patients diagnosed with breast cancer and meningeal carcinomatosismeningeal carcinomatosis, assess the prognostic role of these symptoms at the time of diagnosis, and correlate them with prognostic factors.

**Methods:**

This retrospective study consecutively included breast cancer patients diagnosed with meningeal carcinomatosis. The study collected the symptoms of meningeal carcinomatosis at the time of diagnosis. To assess the prognostic role of symptoms in patients’ overall survival, the Cox regression model was used.

**Results:**

A total of 109 patients between the ages of 26 and 79 were included in the study. The most frequently reported symptoms at diagnosis were headache (42.2%), cranial nerve alteration (31.2%), and nausea (30.3%). The study showed a median survival of 2.4 months, with a 95% confidence interval of 1.7–3.0 months. The only symptom found to be associated with a worse prognosis was the presence of cranial nerve abnormalities, with a hazard ratio of 1.78 (95%CI 1.08–2.95, p=0.024). Conversely, the presence of meningeal hyperenhancement on magnetic resonance was related to a better prognosis, with an hazard ratio of 0.58 (p=0.02, 95%CI 0.36–0.92).

**Conclusion:**

It can be concluded that meningeal carcinomatosis is a serious condition, and the most common symptoms at diagnosis are headache and nausea. Special attention should be given to patients who present with alterations in cranial nerves, as they belong to a subgroup with a poor prognosis.

## INTRODUCTION

Meningeal carcinomatosis (MC) or leptomeningeal metastasis (LM) is the metastatic spread of the primary tumor to the leptomeninges, leading to central nervous system dysfunction. Breast, lung, and melanoma tumors are the most frequent causes of MC among solid tumors in adults^1^.

In patients with metastatic breast cancer, the occurrence of MC varies between 2 and 5% of patients. Previous studies have shown that, in women with breast cancer, invasive lobular carcinoma and cancer positive for the receptor 2 of human epidermal growth factor (HER2) have specific predilections for metastasizing to the leptomeninges^2–5^.

Modern diagnostic methods are being studied for the diagnosis of MC^6^, but the gold standard is still done through the analysis of cerebrospinal fluid (CSF) combined with magnetic resonance imaging (MRI) or computed tomography and symptoms^7^. The most common symptoms leading to the diagnosis are headache, whether or not related to nausea and vomiting, dizziness, altered mental state (confusion, forgetfulness, disorientation, subtle personality changes, and/or lethargy), and cranial neuropathies (when cranial nerves are involved)^8^.

Prognostic factors associated with an MC, such as performance status (PS) (ECOG—"Eastern Cooperative Oncologic Group" or KPS—"Karnofsky performance status"), age, number of previous systemic treatments, molecular profile of the tumor, and biochemical characteristics in a CSF sample, have already been analyzed in previous studies. However, there is no clear consensus on the classification of prognostic risk, and few studies have analyzed the prognostic impact of signs and symptoms in the initial diagnosis of an MC^9,10^.

Therefore, the aim of our study is to describe the main signs and symptoms at the time of diagnosis of an MC and to analyze whether these changes are related to a worse prognosis in these patients.

## METHODS

This is an observational, retrospective study that examined patients with malignant breast cancer diagnosed with MC between 2013 and 2020.

We included consecutive patients with positive cytology for neoplastic cells in CSF or clinical suspicion of LM through the institution's electronic medical chart (TASY—Philips^®^) searched by the Classification of Diseases and Related Health Problems (ICD 10 C50) and disease stage. Of the 3,740 patients with metastatic breast cancer, 109 were included with the diagnosis of LM. In 70 (64.2%) patients, the diagnosis was confirmed by positive cytology in CSF; in 39 (35.8%) based on meningeal enhancement in imaging exams; and in 27 (24.7%), the diagnosis was confirmed by both methods (cytology and imaging exams).

The protocol for this study was reviewed and approved by the institution's ethics committee (IBCC Oncology, CAAE:32154920.8.0000.0072 and protocol 4.069.647), and due to the retrospective nature of the study, the informed consent form was waived.

The study included women with malignant breast cancer over the age of 18 years who had been confirmed to have MC by an imaging test (preferably magnetic resonance) and/or the presence of neoplastic cells in a CSF analysis. Patients with solid tumors of other etiologies or hematological cancer were excluded.

The radiological criterion used to diagnose MC was a typical MRI finding that suggests a diagnosis of MC, the leptomeningeal enhancement. The CSF was analyzed by direct cytological examination to detect neoplastic cells.

We collected symptoms at the initial suspicion of MC and at least 30 days before the MC confirmation. Two independent researchers used clinical judgment to evaluate whether the symptoms were related to MC or related to cancer treatment toxicity.

### Statistical analysis

Categorical variables were described using frequency distribution, and continuous variables using mean and range values.

The Cox regression model was used to assess the prognostic role of symptoms.

We considered as potential confounders age (as a continuous variable) and the PS of the patients according to the ECOG, as a dichotomous variable: 0–1 vs. ≥2.

We used two Cox models. The first included the patient's symptoms at the time of diagnosis, and the second included changes in CSF analysis and changes in imaging tests. We used a statistical significance level of 0.05 to consider the sign/symptom as a factor associated with prognosis.

Sensitive analyses were carried out, including the following variables in the models above: chemotherapy previous line numbers, breast cancer molecular profile, and radiotherapy treatment.

## RESULTS

The study included 109 patients aged between 26 and 79, the majority of whom (69.7%) had hormone receptor-positive breast cancer and HER2 negative, followed by triple negative tumors (18.4%) and HER2 positive (11.9%). A total of 88 patients (80.7%) had ductal histology breast cancer. Most patients had a good PS (ECOG 0–1) and had received 2 or more lines of systemic treatment. Regarding the method used to diagnose MC, approximately 64% of the patients had meningeal hyperenhancement as evidenced by MRI, and 60.5% had neoplastic cells in the CSF analysis. The majority of patients (78.9%) had bone metastases, 58.7% had visceral metastases, and 19.3% had macroscopic lesions in the central nervous system (CNS). No patient had LM at the time of breast cancer diagnosis, and seven patients (6.4%) had recurrence after previous curative intent treatment. The full description of the patients’ characteristics is summarized in Table 1.

**Table 1:** Patients’ clinical characteristics.

Characteristics		All patients (n=109)
Age—years (average, range)		55.6 (26–79)
Histology		
	Ductal	88 (80.7%)
	Lobular	14 (12.9%)
	Other	7 (6.4%)
Molecular type		
	Luminal A/B	76 (69.7%)
	HER2 positive	5 (4.6%)
	Triple negative	20 (18.4%)
	Hormone receptor positive, HER2 positive	8 (7.3%)
Diagnostic method for meningeal carcinomatosis		
Meningeal enhancement in imaging exams		
	Absent	39 (35.8%)
	Present	70 (64.2%)
Presence of neoplastic cells in the cerebrospinal fluid		
	Absent	43 (39.5%)
	Present	66 (60.5%)
	Patients with positive imaging and cerebrospinal fluid	27 (24.7%)
Metastatic sites		
	Bones	86 (78.9%)
	Visceral	64 (58.7%)
	Macroscopic lesions in the CNS	21 (19.3%)
ECOG		
	0–1	67 (61.5%)
	2–4	42 (38.5%)
Previous lines of chemotherapy		
	Up to one line	34 (31.2%)
	Two or more lines	75 (68.8%)

CNS: central nervous system; ECOG: Eastern Cooperative Oncologic Group.

The symptoms present at the time of diagnosis of MC were headache, seizure, mental confusion, nausea, motor deficit, cranial nerve alteration, and dizziness, the most common being headache, nausea, and cranial nerve alteration. The majority of patients had one symptom at the diagnosis of LM (64, 58.7%), followed by two symptoms (31, 28.4%), three symptoms (11, 10.1%), and four symptoms (3, 2.8%).

The median survival of the patients with LM was 2.4 months (95%CI 1.7–3.0). Using age and PS as confounders, we observed that cranial nerve change was associated with a higher risk of death (HR 1.78, p=0.024, 95%CI 1.08–2.95). The median survival of those with and without cranial nerve change was, respectively 2.1 months (95%CI 0.9–3.0), and 2.5 months (95%CI 1.8–4.1).

Headache, nausea, dizziness, seizures, mental confusion, and motor deficits were not associated with a worse prognosis, as described in Table 2.

**Table 2:** Symptoms of meningeal carcinomatosis and relation to risk of death.

Symptoms	All patients (n=109)	Adjusted hazard ratio*		
	n (%)	Hours	95%CI	p-value
Headache	46 (42.2%)	1.26	0.77–2.06	0.340
Seizure	13 (11.9%)	1.82	0.94–3.51	0.077
Mental confusion	9 (8.3%)	1.80	0.81–3.99	0.145
Nausea	33 (30.3%)	0.98	0.63–1.55	0.968
Motor deficit	22 (20.2%)	0.86	0.48–1.53	0.605
Cranial nerve deficit	34 (31.2%)	1.78	1.08–2.95	0.024
Dizziness	16 (14.7 %)	1.27	0.71–2.27	0.415

CI: confidence interval.

* Age and performance status as potential confounders.

In the CSF analysis, the number of neoplastic cells or the concentration of proteins and glucose were not related to a worse prognosis, and the presence of meningeal hyperenhancement on the MRI seems to be related to a better survival rate (HR 0.58, p=0.02, 95%CI 0.36–0.92) as described in Table 3. The median survival of those with meningeal hyperenhancement on the MRI was 2.9 months (95%CI 2.1–4.4), and among those without hyperenhancement, the median survival was 1.0 (95%CI 0.7–1.9).

**Table 3:** Cerebrospinal fluid analysis parameters, imaging findings, and relation to mortality.

Parameters	Patients (n=109)	Adjusted hazard ratio*		
	n (%)	Hours	95%CI	p-value
Positive cytology for malignant neoplastic cells	24.7% (0–100%)	1.00	0.99–1.03	0.577
Mean proportion (range) n (%)	66 (60.6%)			
Elevated cerebrospinal fluid proteins**	74 (67.9%)	1.22	0.79–81.8	0.367
Low glucose concentration in cerebrospinal fluid***	41 (37.6%)	1.24	0.78–1.88	0.367
Leptomeningeal enhancement by MRI	70 (64.2%)	0.58	0.36–0.92	0.02

CI: confidence interval; MRI: magnetic resonance imaging.

* Age and performance status as potential confounders.

** Values ≥45 mg/dL according to local lab reference.

*** Values ≤50 mg/dl according to local lab reference.

Sensitive analyses, including the previous treatment lines number and the molecular subtypes (HER2 positive/triple negatives and luminal A/B) in the Cox regression models, did not change the prognostic role of the cranial nerve deficit (adjusted HR 2.2, 95%CI 1.29–3.74, p=0.004) or the leptomeningeal enhancement by the MRI (adjusted HR 0.51, 95%CI 0.32–0.83, p=0.007).

In total, 40 patients (36.7%) were treated with cranial or spinal radiotherapy. Even after the adjustment of the Cox model including previous use of radiotherapy, the meningeal enhancement by cranial MRI remained as a good prognostic factor (adjusted HR 0.62, 95%CI 0.38–0.99, p=0.047).

## DISCUSSION

The signs and symptoms of MC depend on the site affected by the neoplastic cells; thus, due to multifocal involvement, the presentation is not specific. Clinical findings are often attributed to cranial and/or spinal nerve dysfunction, increased intracranial pressure, or meningeal irritation^11^.

In this study, headache and nausea were the main symptoms observed, which is in line with other series of patients with this clinical condition. In a series of 150 patients published by Clarke et al., the most common symptoms were headache, nausea, vomiting, cerebellar changes, leg weakness, and low back pain^12^.

The presence of neurological deficits in cranial nerves was the only symptom associated with a worse prognosis in our series. Most of the published studies on prognostic factors of MC have evaluated epidemiological or molecular factors and have not addressed the prognostic role of patients’ signs and symptoms.

Azevedo et al. analyzed the association between clinical characteristics and prognostic factors in 60 women with breast cancer with MC and observed that histological grade, PS, and liver metastases were related to a worse outcome in these patients^13^.

A US study published in 2015 retrospectively examined patients diagnosed with MC of various etiologies (not just breast) for 15 years and concluded that age, gender, previous cranial radiotherapy, MC at the time of the initial primary diagnosis, cranial or spinal MC, and histologic type had no significant effect on overall survival. The same study showed that the presence of neurological symptoms leading to an imaging test and ultimately to a diagnosis of MC had a trend effect towards a worse median survival (2.0 vs. 3.8 months, although without reaching statistical significance, p=0.06), i.e., indirectly pointing to a relationship between symptoms and worse outcomes^14^.

Hyun et al., published a study on the clinical experience of 519 patients diagnosed with MC, in which the primary breast tumor was identified in 96 patients, surpassed only by lung cancer, with 334 patients. The most commonly encountered symptoms were headaches and cranial and spinal nerve alterations. With regard to the median survival rate, there was no difference between the two sites, with an overall survival of 3 months. The authors describe that patients with good PS or protein within the normal range in the CSF showed a favorable prognosis^15^.

The proportion of neoplastic cells present in the CSF, as well as low glucose and high protein levels, was not related to a worse prognosis. In recent updated guidelines for the diagnosis, management, and follow-up of MC, Rhun et al., describe the most common changes found in the CSF at the diagnosis of MC. These findings include increased opening pressure (>200 mm H_2_O), increased leukocyte count (>4 per mm^3^), elevated protein (>500 mg/mm^3^), and decreased glucose (<600 mg/l)^16,17^. These findings are commonly found but are not present in all patients and are not validated as prognostic factors.

In our series, meningeal hyperenhancement found on MRI, on the other hand, is related to better survival; the opposite is observed in other series, where changes seen on MRI can be related to worse outcomes^18–20^. Unlike the other series, we considered MRI alterations to be the meningeal hyperenhancement characteristic of MC and not macroscopic intraparenchymal lesions, which may explain the divergent results. Another point to consider is that meningeal hyperenhancement may motivate the treating doctor to carry out radiotherapy more often (40 of the 109 patients included underwent radiotherapy) so that the treatment could justify the better prognosis. To analyze this interaction, we carried out a sensitive analysis including the use of radiotherapy in the Cox model. Despite the adjustment by the radiotherapy treatment, the meningeal hyperenhancement in MRI remained as a good prognostic factor (adjusted HR 0.62, 95%CI 0.38–0.99, p=0.047), which reassures the finding of the initial model. However, the focus of our study did not consist of analyzing the role of radiotherapy in these patients, which could be the focus of future studies.

Our aim was to assess the prognostic role of signs and symptoms at the time of a diagnosis of an MC. We used age and PS as potential confounders in order to prevent the effects of the symptoms of these two variables, known to be related to a worse prognosis in most cancers, including in patients with MC^13–15^, from interfering with the effect of the association between symptoms and mortality.

We included in the sensitive analyses the molecular profile and previous number of treatment lines, two factors related to a worse prognosis in breast cancer, and we did not find divergent results in the initial models, which reassures the finding that cranial nerve alterations are a bad prognostic factor and the meningeal hyperenhancement in cranial MRI is a good prognostic factor.

Our study has some limitations, such as the fact that the data were retrospective and the symptoms were collected from medical records at the moment of the meningeal carcinomatosis. It is possible that only the most intense symptoms were recorded, and other milder symptoms were not. Another point is that we classified the changes in cranial nerves as a whole, not specifying which nerve was altered, besides the fact that these are data from a single center, which limits the generalization of the findings to other services. However, it is one of the broader series of MC cases in cancer.

## CONCLUSION

We sought to assess the signs and symptoms at the time of diagnosis of an MC and whether these were related to worse outcomes. We conclude that altered cranial pairs are related to worse outcomes and that special attention should therefore be paid to these patients at diagnosis. On the other hand, changes seen on MRI may be related to better outcomes, which could play a role in future studies.

## Data Availability

The datasets generated and/or analyzed during the current study are available from the corresponding author upon reasonable request.
